# A preliminary investigation of the relationship between ^18^F-FDG PET/CT metabolic parameters and prognosis in angioimmunoblastic T-cell lymphoma

**DOI:** 10.3389/fonc.2023.1171048

**Published:** 2023-06-15

**Authors:** Lanping Hu, Nana Luo, Lei Li, Dasheng Qiu, Xiaoyan Hu

**Affiliations:** Department of Nuclear medicine, Hubei Cancer Hospital, The Seventh Clinical School Affiliated of Tongji Medical College, Huazhong University of Science and Technology, Wuhan, Hubei, China

**Keywords:** Angioimmunoblastic T-cell lymphoma, PET/CT, metabolic parameters, prognosis, ^18^F-FDG

## Abstract

**Purpose:**

The goal of the study was to determine the prognostic significance of metabolic parameters in baseline ^18^F-FDG PET/CT images obtained from patients with angioimmunoblastic T-cell lymphoma (AITL).

**Methods:**

Forty patients with pathologically diagnosed AITL who had baseline ^18^F-FDG PET/CT between May 2014 and May 2021 were assessed as part of this study. Maximum standardized uptake value (SUVmax), total lesion glycolysis (TLG), and total metabolic tumor volume (TMTV) were obtained and analyzed. In addition, many relevant features were evaluated, including sex, age, staging, International Prognostic Index (IPI), prediction index for T-cell lymphoma (PIT), Ki-67, and so on. Estimates of progression-free survival (PFS) and overall survival (OS) were determined using the log-rank test and Kaplan-Meier.

**Results:**

The median follow-up was 30.2 months (interquartile range 9.82-43.03). Throughout the follow-up period, 29 (72.5%) deaths occurred and 22 (55.0%) patients made progress. The rates for 2- and 3-year PFS were 43.6% and 26.4%, respectively. The 3- and 5-year OS were 42.6% and 21.5%. For TMTV, TLG, and SUVmax, the cut-off values were 87.0 cm3, 711.1, and 15.8, respectively. Poorer PFS and OS were substantially correlated with high SUVmax and TLG. An increased TMTV suggested a shorter OS. TLG performed independently as OS predictors in multivariate analysis. The risk score for predicting the prognosis of AITL includes the TMTV, TLG, SUVmax, and IPI scores, with 4.5 for TMTV, 2 for TLG, 1.5 for IPI scores, and 1 for SUVmax. Three risk categories of patients with AITL had 3-year OS rates of 100.0%, 43.3%, and 25.0%, respectively.

**Conclusion:**

Baseline TLG was a strong predictor of OS. Here a new prognostic scoring system for AITL based on the clinical indicators and PET/CT metabolic parameters was constructed, which might make stratification of prognosis easy and also help to individualize treatment.

## Introduction

Angioimmunoblastic T-cell lymphomas (AITL), a distinct subtype of peripheral T-cell lymphomas (PTCL), account for between 18 and 36% of PTCL (1). Uncertainty surrounds its pathophysiology. Common T-cell antigens including CD2, CD3, CD5, and CD4 were also strongly expressed in AITL, pointing to the presence of T follicular helper (TFH) markers. TFH cells were also demonstrated to be the counterpart cell type to AITL (2–4). Many studies have suggested that immunological abnormalities brought on by viral infections (e.g. Epstein-barr virus) may be related to the pathophysiology (5, 6). The majority of AITL patients are elderly; at the time of diagnosis, most are already in clinical stages III or IV and don’t present any symptoms in the early stages. Previous studies found OS was approximately 32% at 5 years (7). Previous studies have shown that AITL with poorer prognosis may be associated with overexpression of some specific genes such as IDH2, TET2, FYN, and CD2 (8, 9). For the purpose of directing treatment plans, it is essential to identify which patients would relapse earlier at the time of diagnosis. The prognosis of lymphoma is closely related to multiple factors such as age, stage, symptoms, and physical status. According to these prognostic influencing factors, different prognostic scoring systems have been established for different pathological types of lymphoma. The basis for determining the prognosis of PTCL in clinical and research settings is the International Prognostic Index (IPI), which includes five markers of age, clinical stage, physical condition, lactate dehydrogenase (LDH), and extra-nodal involvement. Based on the IPI score, the prediction index for T-cell lymphoma (PIT), which takes into account bone marrow involvement, age, physical condition, and LDH, was created. However, due to the heterogeneity of AITL, these two indices remain insufficient for early risk stratification (10). Only a few studies have particularly focused on prognostic prediction models for patients with AITL up to this point, such as the AITL prognostic index and the AITLI model, although it is uncertain how applicable these new AITL prognostic models will be in the future (11–14). Therefore, an effective prediction model for AITL prognosis is yet to be researched and developed.

Functional imaging and anatomical imaging are combined in PET/CT with ^18^F-FDG as the tracer, which is currently often utilized in clinical practice for lymphoma staging, determining effectiveness, and prognostic evaluation. It has been shown that tumor load before initial treatment is of significant value in predicting prognosis. To assess the tumor load, PET/CT can quantify the volume of all positive lesions and determine the intensity of ^18^F-FDG uptake within the lesion. The tumor metabolic volume (MTV) and total focal glycolysis (TLG) of high-uptake lymphomas such as diffuse large B-cell lymphoma (DLBCL), peripheral T-cell lymphoma (PTCL), and Hodgkin lymphoma (HL) can help predict patient survival (15, 16). Since there are currently few studies on the use of PET/CT metabolic parameters to assess AITL prognosis, the goal of this study was to determine the predictive value of PET/CT multiparametric indices for patient prognosis prior to initial treatment.

## Materials and methods

### Patients

Between May 2014 and May 2021, a total of 60 patients with AITL had baseline ^18^F-FDG PET/CT evaluation; 40 patients were subsequently recruited after receiving full clinical follow-up and imaging data. 18 years of age or older, lymph node biopsy performed in accordance with 2008 WHO criteria to determine the diagnosis of AITL, patients who underwent ^18^ F-FDG PET/CT prior to receiving any treatment, refraining from using a colony-stimulating factor, glucocorticoids, or any other drugs that promote extramedullary hematopoiesis one week before imaging, and positive lesions on PET scans were the inclusion criteria. The following were listed as exclusion criteria: (1) prior or concurrent malignancies; (2) insufficient clinical, follow-up, and imaging data; (3) poor image quality; (4) severe cardiovascular disease; and (5) infection or chronic inflammation in the acute phase. Sex, age, staging, initial symptoms, ECOG-PS, bone marrow biopsy, IPI, PIT, LDH, beta 2-microglobulin (β2-MG), and Ki-67 labeling index were among the clinical and pathological parameters that were gathered and evaluated. 80% of patients were treated with CHOP or ECHOP chemotherapy regimens. The hospital ethics committee gave its approval for the trial, and patients gave their informed consent.

### Instruments and methods

A GE Discovery STE PET/CT scanner was used to generate ^18^F-FDG PET/CT pictures, and an American-made GE cyclotron was used to autonomously synthesize ^18^F-FDG. All had radiochemical purity levels above 95%. Prior to receiving FDG injections, patients fasted for over 6 hours. Before the test, a tri-directional tube was used to inject ^18^F-FDG (5.5 MBq/kg) intravenously, and the patient was given an hour to rest. Whole-body PET/CT imaging consisted of a PET scan of the entire body (2 minutes for each bed, totaling 6–8 beds) and a CT scan (200mA and 120 kV). PET scan adopted 3D acquisition. The body was scanned from the top of the skull to the middle of the thigh bone, and if necessary, the extremities. The ordered subsets expectation maximization (OSEM) approach was used to reconstruct the PET image, and the CT scanning data was used to adjust for image attenuation. The conventional approach was used to reconstruct the CT picture, and the layer thickness was 3.75mm. For frame-to-frame image parallel fusion presentation on Xeleris and AW workstations, PET and CT images were sent.

### Image analysis

All PET/CT images were outlined and analyzed using Advantage Workstation 4.6 by two experienced nuclear medicine diagnosticians who were blinded to the patient’s clinical outcome, with reference to a third nuclear medicine physician (associate director and above) for decision in case of disagreement. Semi-automated measurements were used to depict the volume of the area of interest (VOI) and determine lesion boundaries, and the lesion’s extent was then carefully adjusted to rule out inflammatory or physiological uptake in the brain, urinary system, and intestine. All involved lymph nodes and extra-nodal lesions were included. Thymus, nasopharynx, pharyngeal lymphatic ring, and spleen were intra-nodal lesions. Spleen SUVmax to liver background ratio greater than 1.5 or spleen longitudinal diameter greater than 13 cm was considered as splenic infiltration. Using the 41% SUVmax threshold approach, MTV was computed in accordance with the guidelines provided by the European Society of Nuclear Medicine. Only when bone marrow involvement was verified by BMB were volumetric measures taken. TLG was calculated by adding the products of the MTV and SUV averages of all lesions, and TMTV was calculated by adding the volumes of all hyper-metabolic lesions.

### Statistical analysis

SPSS 23. 0 software (IBM, Chicago, IL, USA) and GraphPad Prism version 9.0 software (San Diego, CA, USA) were used to conduct the statistical analysis and drawing. Continuous variables were represented by their median or mean ± standard deviation, whereas categorical variables were portrayed as counts. The Pearson chi-squared test or Fisher’s exact test was used to examine differences between subgroups. Receiver-operating characteristic (ROC) analysis was used to determine the ideal critical values for PET/CT metabolic parameters. PFS and OS were calculated using the log-rank test and Kaplan-Meier survival curves. The Cox proportional hazards regression model was utilized for multivariate survival analysis. Each relevant prognostic factor in the univariate analysis was given a N score, based on its HR value, to calculate the AITL prognostic score. Each factor’s HR value was first rounded to obtain an integer and then divided by two to obtain the N score. For example, if HR is equal to 5.6, then N is equal to half of 6, which is equal to 3. The sum of N for each factor was defined as the AITL risk score. *p*<0.05 was considered to be statistically significant.

## Results

### Patients

This study involved forty patients. The average age of the patients was 60.3 years old, and the ratio of males to females was 3 to 1. At initial diagnosis, 36 (90.0%) patients were in stage III or stage IV, and 4 (10.0%) patients were in stage II. Twenty-five (62.5%) patients had extranodal involvement: skin (16 cases), parotid glands (14 cases), lungs (6 cases), bone marrow (5 cases), liver (2 cases), and gastrointestinal tract (1 case). In addition, splenic infiltration was present in 18 cases (45.0%). LDH greater than 400u/L and β2-MG greater than 3mg/L were considered elevated. **Table 1** displays the clinical features of the patients.

**Table 1:** Characteristics of patients.

Characteristics	No. (%)
Sex/male	30 (75.0)
Age, mean (range, year)	60.25 (33-80)
B symptoms/yes	22 (55.0)
Ann Arbor stage	
II	4 (10.0)
III	20 (50.0)
IV	16 (40.0)
ECOG-PS >1	13 (32.5)
Elevated LDH	30 (75.0)
Elevated β2-MG	28 (70.0)
BMB positive	5 (12.5)
Extanodal involvement/yes	25 (62.5)
Extranodal sites >1/yes	14 (35.0)
IPI	
0-1	5 (12.5)
2-3	22 (55.0)
4-5	13 (32.5)
PIT	
0	4 (10.0)
1	15 (37.5)
2	11 (27.5)
3	9 (22.5)
4	1 (2.5)

ECOG-PS, Eastern Cooperative Oncology Group Performance Status; LDH, lactate dehydrogenase; β2-MG, β2-microglobulin; BMB, bone marrow biopsy; IPI, international prognostic index; PIT, prognostic index for T-cell lymphoma.

### ROC curves analysis of PET/CT metabolic multiparameter and Ki67

The median values of TMTV, TLG, SUVmax, and Ki67 were 249.3 (interquartile range 96.4-527.0) cm^3^, 1300.1 (interquartile range 552.8-2925.0), 14.5 (interquartile range 9.9-23.8), and 55% (interquartile range 40.0-67.5%), respectively. ROC curve analysis was used to determine the cutoff values for SUVmax, TMTV, TLG, and Ki67. If the area under the curve (AUC) < 0.6, the grouping used the median as the cutoff value.

The ROC curve for each factor was outlined by assigning the deceased patients to the positive event group and the remaining patients to the negative event group as of the follow-up cutoff time. The SUVmax, TMTV, and TLG AUCs were, respectively, 0.770, 0.818, and 0.815. The cut-off values for TMTV, TLG, and SUVmax were 87.0 cm^3^ (sensitivity 96.6%, specificity 63.6%, p = 0.002, Youden index of 0.602), 711.1 (sensitivity 79.3%, specificity 72.7%, p = 0.002, Youden index of 0.521), and 15.8 (sensitivity 55.2%, specificity 90.9%, p = 0.009, Youden index of 0.461). Due to Ki67’s AUC being less than 0.6, the median expression of Ki67 (55%) was utilized as the dividing line for grouping.

### Intergroup comparison of clinical characteristics

Patients were divided into groups with high TMTV (>87.0 cm3) and low TMTV (87.0 cm3) values, as well as groups with high TLG (>711.1) and low TLG (711.1) values. Patients with progressive disease or death at the follow-up cutoff were included in the poor prognosis group. High TLG was usually associated with higher Ann Arbor staging (p = 0.011), IPI score (p = 0.002), PIT score (p = 0.005), Ki67 index (p = 0.048), and elevated LDH (p = 0.012). The frequency of a bad prognosis was statistically substantially greater in the high-risk TMTV group than in the low-risk group (p=0.037), while the difference in TLG between patients with a poor and good prognosis was not statistically significant (p=0.082) (**Table 2**).

**Table 2:** Comparisons of clinicopathologic characteristics according to TMTV and TLG.

	No.	TMTV			TLG		
		Low	High	P	Low	High	P
Age, >60/≤60	21/19	5/3	16/16	0.408	6/8	15/11	0.286
Sex, male/female	30/10	4/4	26/6	0.089	10/4	10/6	0.492
B symptoms, yes/no	22/18	4/4	18/14	0.528	7/7	15/11	0.446
Ann Arbor stage, II/III-IV	4/36	3/5	1/31	0.020*	4/10	0/26	0.011*
ECGO-PS, 0-1/2-5	27/13	6/2	21/11	0.479	12/2	15/11	0.071
LDH, elevated/normal	30/10	4/4	26/6	0.089	7/7	23/3	0.012*
β2-MG, elevated/normal	28/12	4/4	24/8	0.170	7/7	21/5	0.049*
BMB, negative/positive	5/35	1/7	4/28	0.694	1/13	4/22	0.418
Extranodal sites>1, yes/no	13/27	1/7	12/20	0.179	4/10	9/17	0.491
IPI, 0-2/3-5	15/25	5/3	10/22	0.112	10/4	5/21	0.002*
PIT, 0-1/2-4	19/21	5/3	14/18	0.290	11/3	8/18	0.005*
Ki67, >55%/≤55%	20/20	4/4	16/16	0.653	4/10	16/10	0.048*
Poor prognosis, yes/no	32/8	4/4	28/4	0.037	9/5	23/3	0.082

*Statistically significant. ECOG-PS, Eastern Cooperative Oncology Group Performance Status; BMB, bone marrow biopsy; IPI, International Prognostic Index; PIT, prognostic index for T-cell lymphoma.

### Survival analysis for clinical and PET/CT metrics

The median follow-up time was 30.2 (interquartile range 9.82 to 43.03) months. PFS and OS times were respectively 10.5 (95% CI 8.4-12.6) and 32.8 (95% CI 24.10-41.51) months in the median. Of these patients, 22 (55.0%) patients experienced disease progression (excluding death) during the course of treatment and 29 (72.5%) died. The rates for PFS at 2 and 3 years were 43.6% and 26.4%, respectively, while the rates for OS at 3 and 5 years were 42.6% and 21.5%, respectively.

**Figure 1:**
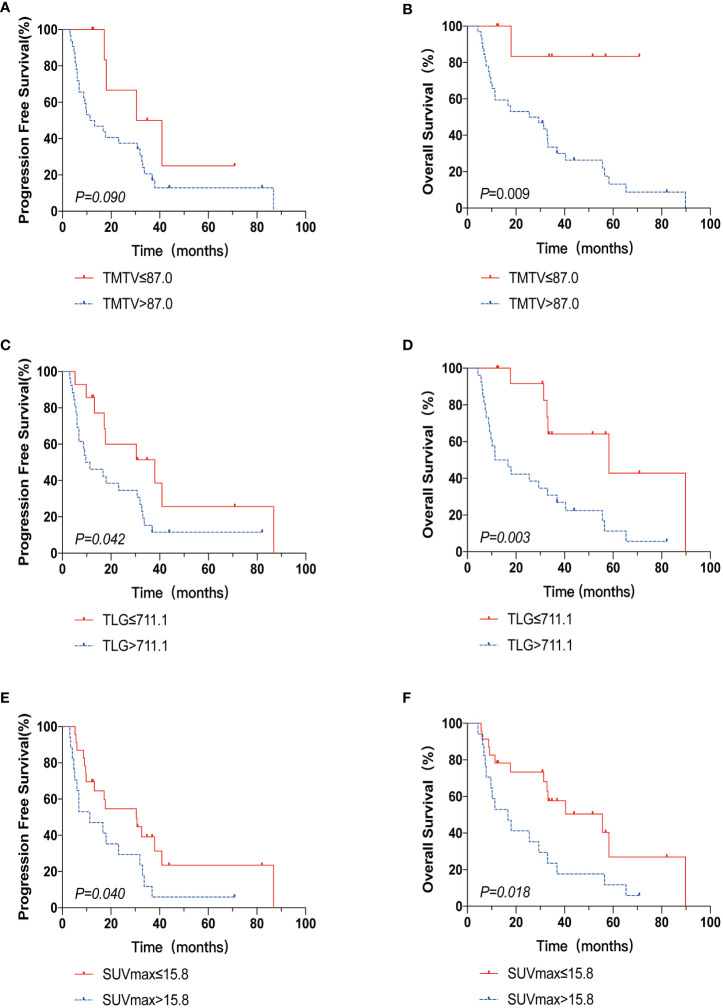
The Kaplan-Meier survival analysis and curves of OS and PFS according to TMTV **(A, B)**, TLG **(C, D)**, and SUVmax **(E, F)**. p value was acquired by the log-rank.

The 2-year PFS rates for the high and low TMTV groups were 37.5% and 66.7%, respectively, according to Kaplan-Meier curves and log-rank testing (p = 0.090). The 3-year OS rates for the high and low TMTV groups were, respectively, 30.1% and 83.3% (p = 0.009). Those with higher TMTV had a median OS of 25.5 months. In the high and low TLG groups, the 2-year PFS rates were 34.6% and 60.0%, respectively (p = 0.042), and the 3-year OS rates were 33.5% and 64.2%, respectively (p = 0.003). The median OS for patients with higher TLG was 11.4 months. In the high and low SUVmax groups, the 2-year PFS rates were, respectively, 29.4% and 54.7% (p = 0.040), and the 3-year OS rates were 23.5% and 57.6%, respectively (p = 0.018). Those with higher SUVmax had a median OS of 16.7 months. Univariate survival analysis revealed that extranodal involvement sites greater than 1, TLG greater than 711.1, and SUVmax greater than 15.8 were risk factors for both PFS and OS, but that high TMTV was only a risk factor for OS and not PFS (**Table 3**; **Figure 1**).

**Table 3:** Univariate analysis for survivals.

	PFS			OS		
	HR	95%CI	P	HR	95%CI	P
Sex (male)	1.72	0.702-4.219	0.235	1.436	0.579-3.562	0.436
Age>60	1.45	0.707-2.961	0.313	1.224	0.581-2.576	0.595
B symptoms	0.67	0.327-1.355	0.262	0.738	0.347-1.567	0.429
Ann Arbor stag (III/IV)	0.04	0.000-2.995	0.143	0.040	0.000-5.222	0.195
ECGO>1	1.51	0.728-3.116	0.270	1.286	0.590-2.804	0.527
Elevated LDH	1.064	0.453-2.498	0.887	1.589	0.588-4.294	0.362
Elevated β2-MG	1.84	0.789-4.264	0.158	1.942	0.784-4.810	0.151
IPI>2	2.20	0.981-4.926	0.056	2.542	1.077-6.003	0.033
PIT>1	1.728	0.836-3.570	0.140	0.195	0.773-3.544	0.195
BMB/positive	1.44	0.707-2.910	0.317	1.222	0.417-3.581	0.715
Extranodal sites >1	2.586	1.224-5.463	0.013	2.502	1.118-5.599	0.026
TMTV>87.0	2.414	0.842-6.921	0.101	8.960	1.216-65.988	0.031
TLG>711.1	2.263	1.005-5.093	0.049	4.007	1.516-10.587	0.005
SUVmax>15.8	2.068	1.017-4.208	0.045	2.413	1.134-5.133	0.022
Ki-67>55%	1.029	0.507-2.091	0.936	0.986	0.468-2.076	0.970

IPI, international prognostic index; PIT, prognostic index for T-cell lymphoma, ECOG-PS, Eastern Cooperative Oncology Group Performance Status; LDH, lactate dehydrogenase; β2-MG, β2-microglobulin, BMB, bone marrow biopsy, SUVmax, maximum standard uptake value; TMTV, total metabolic tumour volume, TLG, total lesion glycolysis.

There was a strong correlation between TMTV and TLG (r = 0.868, p < 0.001) by Spearman’s rank correlation test; therefore, TMTV or TLG were included in the multivariate analysis with other clinical characteristics at p < 0.05 in the univariate analysis, respectively. TLG was a predictive factor for OS independently (HR 3.32, 95% CI 1.080-9.582, p=0.036), while TMTV tended to be an independent OS predictor (p=0.055) (**Table 4**).

**Table 4:** Multivariate analysis for survivals.

	PFS				OS		
	HR	95%CI	P		HR	95%CI	p
TMTV*							
TMTV	2.08	0.687-5.955	0.201	TMTV	7.19	0.957-54.00	0.055
SUVmax	1.82	0.881-3.796	0.105	SUVmax	1.70	0.808-4.985	0.133
Extranodal sites >1	2.08	0.996-4.500	0.061	IPI>2	1.30	0.442-3.673	0.655
				Extranodal sites >1	1.93	0.874-4.663	0.100
TLG*							
TLG	1.74	0.714-4.245	0.223	TLG	3.22	1.080-9.582	0.036
SUVmax	1.51	0.689-3.306	0.303	SUVmax	1.44	0.581-3.547	0.434
Extranodal sites >1	2.23	1.045-4.743	0.038	IPI>2	1.12	0.376-3.316	0.843
				Extranodal sites >1	2.35	0.989-5.598	0.053

PFS, progression-free survival; OS, overall survival; HR, hazard ratio; CI, confidence interval; TMTV, total metabolic tumour volume; TLG, total lesion glycolysis.

*TMTV and TLG were separately incorporated into multivariate survival analysis due to the correlation between TMTV and TLG.

### Construction of AITL risk score and prognostic predictive efficacy

**Figure 2:**
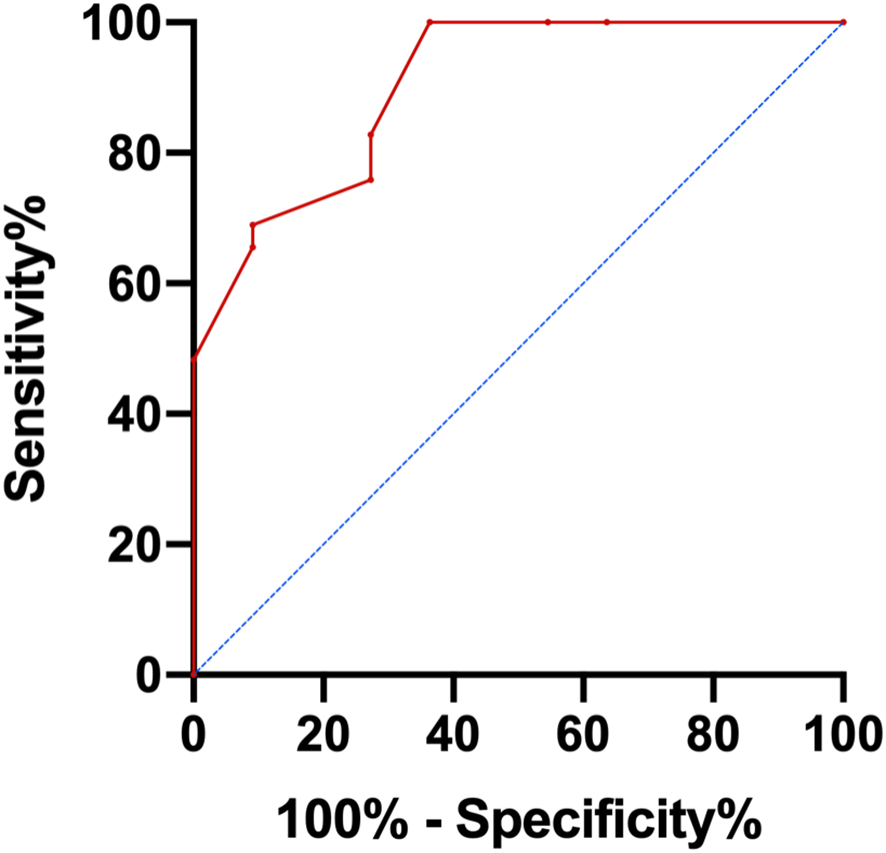
ROC curve analysis of AITL risk score.

According to univariate analysis, TMTV, TLG, SUVmax, IPI score, and more than one extra-nodal involvement site were predictive variables for OS, and extra-nodal involvement sites >1 was an indicator of IPI score, so it was not included in the risk score. N points were allocated to each element in accordance with the AITL risk score methodology, with 4.5 scores for TMTV (HR: 8.960); 2 scores for TLG (HR: 4.007); 1.5 scores for IPI scores (HR: 2.542); and 1 score for SUVmax (HR: 2.413). Therefore, the AITL risk score for this group of patients ranged from 0 to 9. The AUC of the AITL risk score obtained by ROC curve analysis was 0.903 (**Figure 2**).

**Figure 3:**
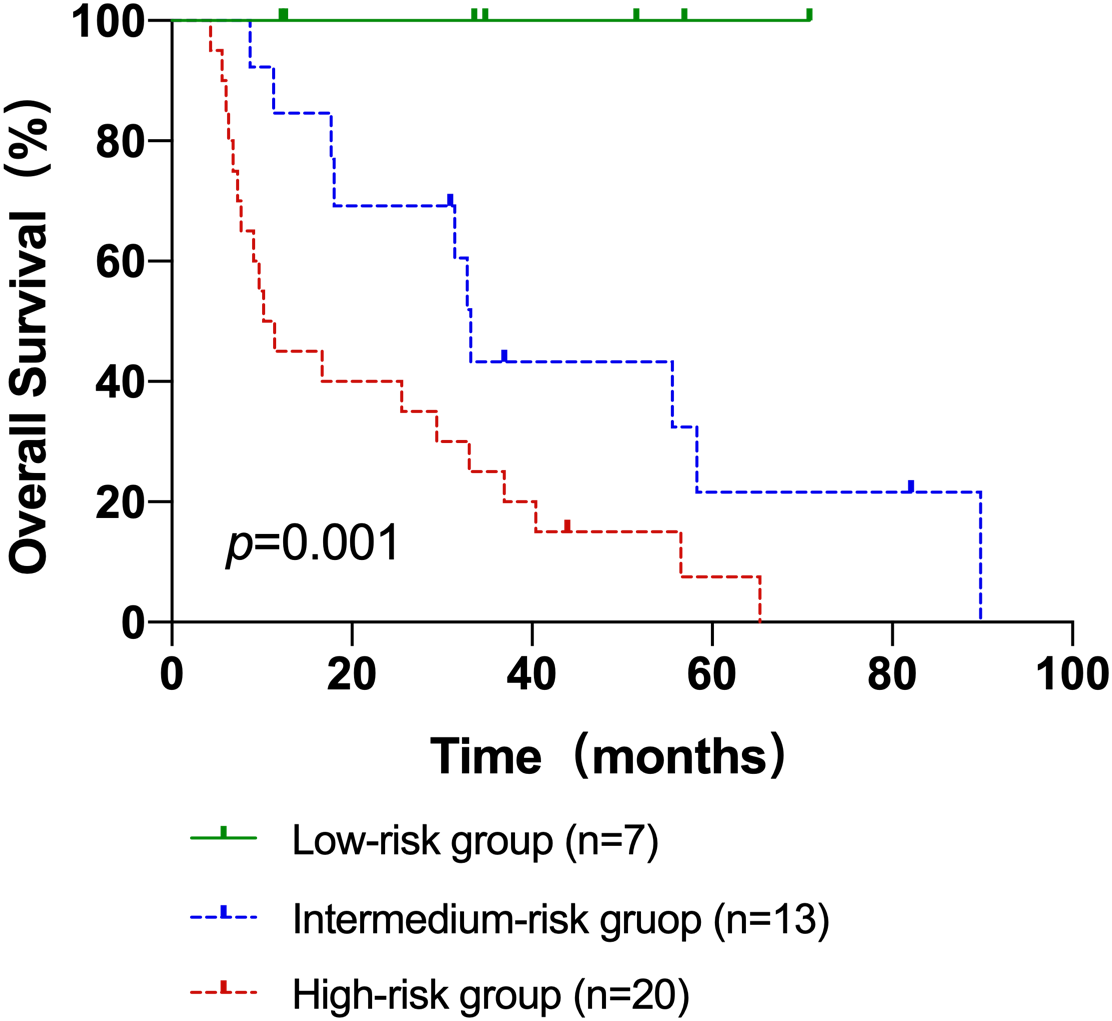
The Kaplan–Meier survival analyses and curves of OS according to the AITL risk scores. p value was acquired by the log-rank test.

**Figure 4:**
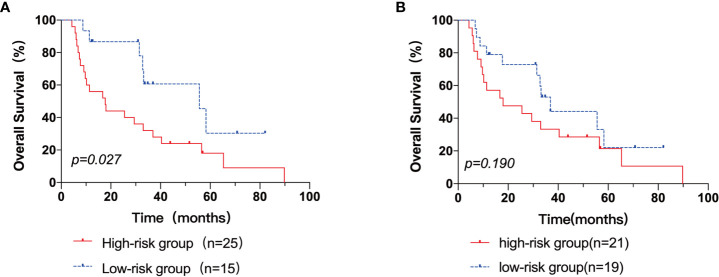
The Kaplan–Meier survival analyses and curves of OS according to the IPI score **(A)** and PIT score **(B)**. p value was acquired by the log-rank test.

Next, patients were classified into three groups depending on their AITL risk scores: low-risk group (score 0–3), medium-risk group (score 4.5–7.5), and high-risk group (score 8–9). In the three groups, the 3-year OS rates were 100.0%, 43.3%, and 25.0%, respectively (p=0.001) (**Figure 3**). Kaplan-Meier survival analysis of IPI scores versus PIT scores showed a significant difference between the low-risk and high-risk groups in the patient groups by IPI (P < 0.05), while AUC = 0.746 was obtained by ROC curve analysis (**Figure 4A**). In the group of patients by PIT, there was no significant difference between the low-risk and high-risk groups (P > 0.05) (**Figure 4B**). These analyses suggest that neither IPI nor PIT showed good prognostic performance in patients with AITL.

## Discussion

### Current status of clinical assessment of prognosis in AITL

A rare form of PTCL, AITL is marked by quick disease progression and a dismal prognosis. A large-scale population-based analysis using the SEER database was carried out by Xu and Liu et al. to elucidate the temporal survival trends and prognostic variables of AITL patients (17). A total of 1207 AITL patients were enrolled in this study, and their respective OS rates at 2, 5, and 10 years were 46.8%, 32.9%, and 21.9%. The 3-year OS rate and 5-year OS rate in our retrospective analysis, respectively, were 42.6% and 21.5%. (95% CI: 24.092-41.508; median OS: 32.8 months). The 5-year survival rate for patients with AITL was less than 40%, according to studies conducted so far (18), and the current investigation supports this conclusion. Patients with AITL have poorer outcomes than patients with aggressive B-cell lymphoma and may relapse early, but a minority of patients are able to survive long-term or even be cured. Hence, it is crucial to accurately determine the prognosis of patients with AITL and to more accurately distinguish between those who are in danger and those who may be able to receive a cure.

Recently, some studies have analyzed the relationship between clinical features and the prognosis of AITL. In a retrospective investigation of 207 AITL patients, Tokunaga et al. discovered that age over 60 years, increased leukocyte and IgA levels, anemia and thrombocytopenia, and more than one site of extra-nodal involvement were important predictive factors for OS, and for PFS, mediastinal lymph node metastasis, increased IgA, and anemia were significant prognostic factors (13). It is still debatable if these clinical signs have any real impact on the prognosis of AITL. The IPI score is widely used as a predictive model for various non-Hodgkin’s lymphomas, and the recently proposed PIT score can also stratify or predict the risk of PTCL. But several investigations have demonstrated that the IPI and PIT were insufficient as indicators of prognosis in individuals with AITL (10, 12, 19, 20).

### The value of ^18^FDG-PET/CT in lymphoma prognosis prediction

^18^F-FDG PET/CT plays an important role in the diagnosis and treatment of malignant lymphoma, and the calculation of TMTV as well as TLG allows the quantification of the total tumor metabolic activity volume. The International Society for Malignant Lymphoma stated that metabolic parameters of ^18^F-FDG PET/CT could be used in the prognostic analysis of lymphoma (15). Several large retrospective or prospective studies have shown that multiple metabolic parameters of PET/CT (including TMTV and TLG) affect the survival prognosis of HL, DLBCL, and T-cell lymphoma subtypes (21, 22). As a result, there is an increasing interest in TMTV and TLG, sometimes in combination with clinical parameters to assess prognosis (23). In order to assess the prognosis of AITL or PTCL, Gong, Cottereau, and Jiang et al. used TMTV and PIT scores. They demonstrated that the two factors together may more effectively predict the prognosis of patients (24–26).

We looked into the prognostic value of a variety of metabolic parameters of pretreatment PET/CT. In multivariate survival analysis, we found that TLG was an independent prognostic factor for OS (HR=3.22, 95% CI%:1.080-9.582, p=0.036), and TMTV showed a trend as an independent predictor of OS (HR=7.19, 95% CI%:0.957-54.00, p=0.55). According to the analysis of PET/CT metabolic characteristics of 56 patients before treatment by Gong et al., TMTV was a single factor affecting PFS and OS in AITL patients (24). In a multicentre retrospective study of 140 patients with PTCL in the lymph nodes, Cottereau et al. found that baseline TMTV was the only independent variable considered significant in PFS and OS (25). Zhou et al. believed that baseline TMTV and TLG were independent predictors of PFS and OS (27). Pak et al. found that TLG elevation was most significant in a multicenter trial involving 36 patients with extranodal NK/T cell lymphoma (28). We hypothesized that this contradiction was due to the high correlation between TLG and TMTV and that including both in a multivariate analysis would lead to an incorrect assessment. Furthermore, it was found that the optimal cut-off values for classifying patients into high- and low-risk populations differed depending on studies, which correlated with the clinical features of the study population (volume range, treatment effect, etc.). This implies that the ideal cut-off values for risk prediction using TMTV and TLG may be unique to certain patient traits, lymphoma subtypes, and treatments.

The most common index for determining the amount of ^18^F-FDG uptake, SUVmax, indicates the tumor’s most aggressive cellular component’s glycolytic metabolism, and studies have linked SUVmax to tumor aggressiveness (29). The predictive significance of SUVmax before therapy is still debatable, though. By using univariate analysis, the current study demonstrated a substantial correlation between SUVmax and prognosis, but it did not serve as an independent predictor of AITL (p>0.05). The findings of Gong and Wang were similar to ours, but there were also differences, which showed no significant prognostic value of SUVmax in both univariate and multivariate analyses (23, 30). The study by Jiang et al. did not find that SUVmax correlated with the prognosis of PTCL (25). The possible reasons we considered were as follow: firstly, SUVmax is susceptible to injection time, blood glucose levels, and partial volume effects. Focal FDG affinity is variable at baseline. A recent study used dynamic changes in SUVmax over the course of treatment to assess the predicted prognosis of lymphoma (31). Second, SUVmax represents only a portion of the volume of FDG uptake in a single lesion, whereas patients with AITL usually have multiple lesions. In addition, due to the large heterogeneity and prognostic differences between the different pathological subtypes of PTCL, the prognostic predictive role of SUVmax in PTCL did not lend itself to direct application to AITL.

### Evaluation value of clinical features for prognosis in AITL

In this study, patients’ clinical characteristics including age, sex, B symptoms, Ann Arbor stage, ECGO score, PIT score, and laboratory clinical indicators (LDH, β2-MG) were not linked with OS. IPI score correlated with OS (HR=2.542, 95%CI: 1.077-6.003, p=0.033), and extranodal involvement>1 was associated with OS and PFS in AITL (PFS: HR=2.586, 95%CI: 1.224-5.463, p=0.013; OS: HR=2.502, 95%CI: 1.118-5.599, p=0.026). Moreover, it served as a standalone predictive factor for PFS (HR = 2.230, 95% CI = 1.045–4.743, p = 0.038). However, the present study did not find the value of the PIT score in AITL prognosis (p>0.05). This contradicts some of the previous studies. On the one hand, it may be because the PIT includes 4 parameters, which has a confounding bias. On the other hand, the number of patients enrolled was small and there was selection bias. Previous studies have also shown that IPI and PIT scores for patients with AITL could not be used to predict survival, and even when they were taken into account in multivariate analyses, they had no appreciable impact on survival rates. The present study hypothesized that the clinical outcome of AITL is not so much a direct complication of tumor proliferation as a result of a severe regulatory disorder of the immune system.

### Construction of an AITL risk score based on clinical indicators and PET/CT metabolic parameters

Based on clinical indices and PET/CT metabolic parameters, we innovatively constructed a risk score for prognosis prediction of AITL, including TMTV, TLG, SUVmax, and IPI score, and the prognosis of AITL patients was successfully stratified. Three groups of patients with AITL were created: a low-risk group, an intermediate-risk group, and a high-risk group. Patients in the low-risk group had considerably greater OS rates than those in the intermediate and high-risk groups, according to the Kaplan-Meier survival analysis. The three groups’ respective 3-year OS rates were 100.0%, 43.3%, and 25.0% (2 = 14.639, p0.001). The novel prognosis scoring system has the ability to more accurately predict the prognosis of AITL, even if the IPI score and SUVmax were not independent prognostic markers for AITL and TMTV only showed a trend as an independent predictor of OS in this study (p>0.05). This study was unable to properly explore the effect of altering and shifting treatment patterns on the prognosis of patients with AILT due to the small sample size. This prognostic prediction score should be validated in future large sample and prospective studies, providing valuable information to optimize treatment decisions to benefit more AITL patients.

### The limitation of the previous studies and differences of this study

Most previous studies have only explored the prognostic value of PET/CT metabolic parameters by retrospective analysis or combined with clinical prognostic scores to construct prognostic models (24, 30). Due to the rarity of AITL, most studies currently only explored the prognostic value of PET/CT in PTCL as a whole, with AITL as only a subset of cases (27, 31, 32). Most studies on the prognosis of FDG-PET/CT in AITL were small sample studies, which means that unrecognized bias and the presence of overfitting cannot be avoided. The long time span of patients enrolled in the study and the inconsistency of first-line chemotherapy may cause bias, which is the limitation of this study. Here our study developed a new prognostic scoring system specifically designed for AITL based on clinical indicators and PET/CT parameters, which clearly defined risk groups in AITL patients and identified patients with relatively better prognosis, as compared to the existing prognostic models. Hence this novel prognostic model specially designed for AITL may facilitate risk-based stratification and therapy.

### Future prospects

External data validation would be needed to verify the effectiveness of the new scoring system in the future. Several studies have verified the efficacy of intermediate FDG-PET as a prognostic indicator in lymphoma. 95 patients with PTCL participated in a recent study by Casulo et al. that examined intermediate FDG-PET/CT. This study showed that clinical outcomes could be predicted by measuring metabolic activity using intermediate FDG-PET/CT in PTCL (32). The value of interim FDG-PET for assessing the prognosis of AITL can be further explored in the future. Moreover, to combined predictive model of ^18^F-FDG and clinicopathological characteristics is a promising research direction. More biomarkers need to be investigated in future studies and applied to the new AITL prognosis prediction model.

## Conclusion

We concluded that baseline TMTV, TLG, and SUVmax were independent predictors of worse outcomes in AITL, while baseline TLG was an independent predictor of OS. We have developed a new prognostic scoring system specifically designed for AITL based on clinical indicators and PET/CT parameters, which may assist in clinical decision-making for AITL patients in clinical practice and also provide a basis for future research.

## Data availability statement

The raw data supporting the conclusions of this article will be made available by the authors, without undue reservation.

## Ethics statement

The studies involving human participants were reviewed and approved by Hubei Cancer Hospital. The patients/participants provided their written informed consent to participate in this study.

## Author contributions

LH participated in the design of the study, carried out analysis and interpretation of data, and drafted the manuscript; final approval of the version to be published and agree to be accountable for all aspects of the work. LH, NL, and LL involved in image analysis, participate in the discussion of the result of the part and final approval of the version to be published. DQ and XH gave conception and design of the study, participated in the image analysis, participate in the discussion of the results analysis, and approved the final submission. All authors read and approved the final manuscript. All authors contributed to the article and approved the submitted version.
